# Ab Initio Study of Fine and Hyperfine Interactions in Triplet POH

**DOI:** 10.3390/molecules27010302

**Published:** 2022-01-04

**Authors:** Luca Bizzocchi, Silvia Alessandrini, Mattia Melosso, Víctor M. Rivilla, Cristina Puzzarini

**Affiliations:** 1 Scuola Normale Superiore, Piazza dei Cavalieri 7, 56126 Pisa, Italy; luca.bizzocchi@unibo.it (L.B.); silvia.alessandrini@sns.it (S.A.); 2 Dipartimento di Chimica “Giacomo Ciamician”, Università di Bologna, Via F. Selmi 2, 40126 Bologna, Italy; mattia.melosso2@unibo.it; 3 Scuola Superiore Meridionale, Università di Napoli Federico II, Largo San Marcellino 10, 80138 Naples, Italy; 4 Centro de Astrobiología (CSIC-INTA), Ctra. de Ajalvir Km. 4, Torrejón de Ardoz, 28850 Madrid, Spain; vmrivilla@gmail.com; 5 INAF—Osservatorio Astrofisico di Arcetri, Largo E. Fermi 5, 50125 Florence, Italy

**Keywords:** POH radical, fine splitting, hyperfine structure, quantum chemistry, astrochemistry

## Abstract

Phosphorous-containing molecules have a great relevance in prebiotic chemistry in view of the fact that phosphorous is a fundamental constituent of biomolecules, such as RNA, DNA, and ATP. Its biogenic importance has led astrochemists to investigate the possibility that P-bearing species could have formed in the interstellar medium (ISM) and subsequently been delivered to early Earth by rocky bodies. However, only two P-bearing molecules have been detected so far in the ISM, with the chemistry of interstellar phosphorous remaining poorly understood. Here, in order to shed further light on P-carriers in space, we report a theoretical spectroscopic characterisation of the rotational spectrum of POH in its ${}^{3}$A${}^{\prime \prime }$ ground electronic state. State-of-the-art coupled-cluster schemes have been employed to derive rotational constants, centrifugal distortion terms, and most of the fine and hyperfine interaction parameters, while the electron spin–spin dipolar coupling has been investigated using the multi-configuration self-consistent-field method. The computed spectroscopic parameters have been used to simulate the appearance of triplet POH rotational and ro-vibrational spectra in different conditions, from cold to warm environments, either in gas-phase experiments or in molecular clouds. Finally, we point out that the predicted hyperfine structures represent a key pattern for the recognition of POH in laboratory and interstellar spectra.

## 1. Introduction

Phosphorus (P) is essential for all living organisms [1]. Molecules containing this element play fundamental roles in genetic information storage and transfer, metabolic processes, and compartmentalisation. Furthermore, phosphorus is one of the basic constituent of biomolecules, such as ribonucleic acid (RNA), deoxyribonucleic acid (DNA), adenosine triphosphate (ATP), and phospholipids. Laboratory prebiotic experiments have also stressed the role of P-bearing species as catalysts and chemical buffers in the formation of RNA nucleotides [2].

In recent years, the biogenic relevance of P has attracted much attention from the astrobiological/astrochemical community since prebiotic chemicals might have been carried to early Earth through extraterrestrial delivery (see, e.g., Ref. [3]). For example, it has been proposed that meteorites [4] and comets [5] might have provided significant amounts of P, contained in minerals, such as schreibersite, or in simple molecular species, respectively. To date, only two P-bearing molecules have been identified in the interstellar medium (ISM), i.e., the gas of the molecular clouds that forms new stars and planets [5,6,7,8,9,10,11,12]: phosphorus nitride (PN) and phosphorus oxide (PO). To attain a better understanding of the phosphorous chemistry in the ISM and to ascertain what are its main carriers in space, the search for new P-bearing species is needed. Molecules containing the P–O bond are particularly relevant since it is the basic building block of the phosphate moiety, which is an essential constituent of biomolecules. This prompted us to investigate the POH radical.

The chemical composition of the ISM is mainly investigated via the recognition of rotational or vibrational transitions in the spectra observed by ground-based radio telescopes and/or spaceborne facilities [13]. The rotational spectrum is often denoted as a collection of “molecular fingerprints” because it substantially depends on the atomic composition and on the molecular structure [14,15,16,17]. In fact, both PN and PO, as well as about 93% of the molecules detected in Space (see https://cdms.astro.uni-koeln.de/classic/molecules for a comprehensive list.), were undoubtedly identified by rotational spectroscopy techniques [18]. The selectivity of rotational spectroscopy is such that conformers, rotamers, and even different isotopologues are readily well distinguishable [19,20]. However, inferring structural and molecular properties from rotational spectra is not at all straightforward [21]. This is especially true when the target of the study is an unstable molecule, as often happens for species of astrochemical interest (as is also the case of the title radical). In these cases, molecular systems have to be produced in situ by, for example, electric discharge or pyrolysis, thus resulting in a congested spectrum, with many signals originated by interfering species and excited vibrational states. In such a complicated situation, the preliminary assignment of spectral lines in terms of quantum numbers relies on the identification of characteristic patterns due, for example, to the *K* structure or, when present, to fine and hyperfine structures. The latter structures are the consequence of the splitting of rotational energy levels caused by electric and/or magnetic interactions and are particularly important features of the rotational spectrum as they are very distinctive of the electronic arrangement of the molecule [14] and therefore of its conformation [22]. These interactions shape the rotational spectrum, creating unique patterns in terms of both line position and intensity that undoubtedly identify the molecule.

State-of-the-art spectrometers, working in the millimetre/submillimetre-wave regions, possess a resolving power that allows, in many cases, identifying and resolving such hyperfine structures [23,24]. On the other hand, the line-by-line assignment of these complex patterns greatly benefits from the availability of effective theoretical simulations based on accurate quantum-chemical techniques [14,15,17,25]. In fact, even though computational spectral predictions are always affected by uncertainties in the absolute line position, the hyperfine structures can be reproduced with great accuracy once the critical spectroscopic parameters are computed at a suitable level of theory [14,25,26].

In the present paper, an accurate computational strategy has been exploited to characterise the rotational spectrum of a species with a complicated fine/hyperfine structure: the POH radical in its ${}^{3}$A${}^{\prime \prime }$ electronic ground state [27]. This is an extremely challenging system to study from an experimental point of view because of its instability and the possibility of easily rearranging the most stable HPO. POH and HPO are structural isomers that present very different characteristics. In fact, POH in its fundamental state is an open-shell species, with the singlet closed-shell state lying higher in energy; on the contrary, for HPO, the triplet state is less stable than the singlet (ground) state [27,28]. This difference in stability has allowed the experimental characterisation of HPO (${\tilde{X}}^{1}$A${}^{\prime }$), and its rotational parameters have already been reported in the literature [29]. On the contrary, for POH, only a computational study at a reasonable good level is available [27].

Spectroscopic information on POH is of great relevance for astrochemical modelling in view of the aforementioned importance of P-bearing species. Furthermore, this species is a key intermediate in the reaction between the OH radical and atomic P (${}^{4}$S) [30]. While this reaction in the gas phase eventually forms PO and H radicals [30], on icy grain mantels, where phosphorus is believed to be present in a large amount, this mechanism could end up with the reactive desorption of POH, possibly avoiding the lattice-mediated rearrangement to a lower energy form. Because of its two unpaired electrons, the rotational spectrum of POH is expected to be characterised by almost all possible fine and hyperfine interactions, thus making the system of great interest from the computational spectroscopy point of view. In the following, a description of the computational methodology employed for the evaluation of the various spectroscopic parameters is given. In the Section 3 (Results), the characterisation of the POH and HPO species will be reported in terms of energetics and molecular structures. In the same section, we also present various rotational spectra of POH, simulated in different observational environments, from the laboratory to astronomical sources. Finally, the concluding remarks are addressed and discussed in Section 4 (Discussion), where the expected uncertainties for what concerns the rotational parameters of POH are reported.

## 2. Materials and Methods

For a closed-shell species, there are three main interactions that lead to a hyperfine structure. Whenever a molecule contains a nucleus with a nuclear spin (*I*) equal to or greater than 1, the nuclear quadrupole coupling occurs. Another possible interaction is that between the (very weak) magnetic field generated by the rotation of the molecule and the nuclear magnetic field, present when $I\geq \frac{1}{2}$ [31]. The third interaction is the dipolar coupling occurring when the molecule possesses more than one non-vanishing nuclear spin [23]. This already complex picture becomes even more tangled when the species under consideration has one or more unpaired electrons. In such a case, one has to also account for (i) the interaction between the electron spin of the unpaired electron and the magnetic field of the rotating molecule (electron spin-rotation interaction); (ii) the interaction between the spins of two unpaired electrons (electron spin–spin coupling); (iii) the electron spin–nuclear spin coupling, which accounts for isotropic (Fermi contact) and anisotropic terms [32]. All these interactions except for the nuclear quadrupole coupling need to be considered for POH. Among them, those interactions involving electron pin (i.e., those that are characteristic of radicals) are stronger than those of closed-shell species.

In the following, the computational methodology employed in this work is detailed. While the main goal of this work is the spectroscopic characterisation of POH in its fundamental state (${}^{3}$A${}^{\prime \prime }$), HPO has been investigated for comparison purposes considering its triplet state, which is not, however, its electronic ground state.

### 2.1. Molecular Structure

The key point to obtain accurate molecular structures is to reduce the errors associated to quantum-chemical calculations (one- and *N*-electron errors) as much as possible. To get rid of the one-electron error due to the truncation of the basis set, we have employed the so-called CBS+CV composite scheme, thereby exploiting the “gradient” approach [33,34] implemented in the quantum-chemical CFour program package [35]. To limit the *N*-electron error associated to the truncation of the wavefunction model, in the CBS+CV scheme, the CCSD(T) method has been used, where the acronym stands for coupled-cluster (CC) singles and doubles with perturbative treatment of triples [36].

Within the CBS+CV scheme, to recover the error due to the basis-set truncation, extrapolation to the complete basis set (CBS) limit is performed [34]. The energy gradient extrapolated to the CBS limit is given by:

(1) (dECBS(dx=(dE∞(HF−SCF)(dx+(dΔE∞(CCSD(T))(dx,

where (dE∞(HF−SCF)/(dx and (dΔE∞(CCSD(T))/(dx are the gradients obtained using the exponential extrapolation formula by Feller for the HF-SCF energy [37] and the $n^{-3}$ extrapolation expression for the CCSD(T) electron correlation contribution [38], respectively. Since the extrapolation to the CBS limit is performed within the frozen-core (fc) approximation, core-valence correlation effects are considered by adding to the CBS gradient of Equation (1) the corresponding correction, (dΔE(CV)/(dx:

(2) (dECBS+CV(dx=(dE∞(HF−SCF)(dx+(dΔE∞(CCSD(T))(dx+(dΔE(CV)(dx,

with the core-correlation energy contribution being evaluated as the difference of all-electron (ae) and fc CCSD(T) calculations using the same basis set. In the CBS+CV scheme, the correlation-consistent polarised cc-p(wC)V*n*Z basis sets [39,40,41,42] have been employed. For the extrapolation to the CBS limit, we have used n=Q−6 for HF-SCF and n=Q,5 for CCSD(T). For the CV correction, we resorted to the cc-pwCVQZ basis set.

To further improve the structural determination, the contributions due to the full treatment of triple (Δr(fT)) and quadruple (Δr(fQ)) excitations have been considered and obtained at a “geometry” level. This means that the corrections to the structural parameters have been derived by comparing the results of different geometry optimisations. The following differences have then been added to the CCSD(T)/CBS+CV geometrical parameters:

(3) $$\begin{array}{ccc} \Delta r(\mathrm{fT}) & = & r(\mathrm{CCSDT})-r(\mathrm{CCSD}(T))\\ \Delta r(\mathrm{fQ}) & = & r(\mathrm{CCSDTQ})-r(\mathrm{CCSDT})\;, \end{array}$$

where *r* denotes a generic structural parameter. The cc-pVTZ basis set has been used for the fT correction and the cc-pVDZ set for the fQ contribution. The geometry optimisations needed for these higher-order corrections are: fc-CCSDT/cc-pVTZ, fc-CCSD(T)/cc-pVTZ, fc-CCSDTQ/cc-pVDZ, and fc-CCSDT/cc-pVDZ, with CCSDT [43,44,45] and CCSDTQ [46] standing for CC singles, doubles, triples and CC singles, doubles, triples, quadruples, respectively. Overall, the resulting level of theory (CBS+CV + fT + fQ) will be denoted in the text as “CC${}_{\mathrm{best}}$”. For these computations, the CFour program package has been interfaced with MRCC [47].

### 2.2. Spectroscopic Parameters

From the equilibrium structure at the “CC${}_{\mathrm{best}}$” level, the equilibrium rotational constants can be straightforwardly derived. However, the effect of molecular vibrations needs to be taken into account in order to provide an accurate prediction of the vibrational ground-state rotational constants. Such an effect can be conveniently described by the means of the vibrational perturbation theory (VPT). While there are no corrections to the first order in VPT, at the second order (VPT2), we obtain [48]:

(4) $$B_{r}^{\beta }=B_{e}^{\beta }-\sum_{r}\alpha_{r}^{\beta }\left(v_{r}+\frac{d_{r}}{2}\right)\;,$$

where the superscript β denotes the inertial axes, and the sum runs over all normal modes *r*; $\alpha_{r}^{\beta }$ are the so-called vibration–rotation interaction constants. Their computation requires the evaluation of an anharmonic force field, which has been calculated at the ae-CCSD(T)/cc-pwCVTZ level, as implemented in CFour.

Moving from the rigid-rotor approximation to a semi-rigid treatment, in addition to the effect of molecular vibrations, the centrifugal distortion needs to be taken into account. Centrifugal-distortion effects can be conveniently treated by means of VPT2. The evaluation of quartic-centrifugal distortion constants requires the computation of a harmonic force field, which has been performed at the ae-CCSD(T)/cc-pwCVQZ level (again using the CFour program). As a byproduct of the ae-CCSD(T)/cc-pwCVTZ anharmonic force-field calculations, the sextic centrifugal-distortion constants have also been obtained.

The effective Hamiltonian operator for triplet radical species is given by the sum of three terms:

(5) $$\tilde{H}={\tilde{H}}_{\mathrm{rot}}+{\tilde{H}}_{\mathrm{fs}}+{\tilde{H}}_{\mathrm{hfs}}\;.$$

where ${\tilde{H}}_{\mathrm{rot}}$ is the rotational Hamiltonian, which only depends on the aforementioned rotational constants and centrifugal distortion constants. ${\tilde{H}}_{\mathrm{fs}}$ and ${\tilde{H}}_{\mathrm{hfs}}$ are the terms that describe the fine and hyperfine structure, respectively. A detailed account of each single term for the POH radical is reported in Section 3 (Results), while a brief description of the computations required to derive the fine and hyperfine rotational parameters is given in the following.

All hyperfine parameters and the electron spin-rotation tensor have been computed at the ae-CCSD(T)/cc-pwCVQZ level, as implemented in the CFour program, while the electron spin–spin tensor has been evaluated using the multi configuration self-consistent field (MCSCF) method with the DALTON electronic structure program [49,50,51].

The hyperfine electron spin–nuclear spin interaction is described through the hyperfine coupling tensor A, which requires the computation of the spin density matrix [52,53,54]. The electron spin-rotation tensor **ϵ** has been calculated in a perturbative manner as the second derivative of the energy with respect to the electron spin and rotational angular momentum as perturbations, as described in Ref. [55]. Finally, the nuclear spin-rotation tensor has been computed as the second derivative of the electronic energy with respect to the rotational angular momentum and the nuclear spin in conjunction with the so-called rotational London orbitals [56,57,58].

The fine splitting of the rotational lines arising from the interaction between the two unpaired electrons (electron spin–spin interaction) was also considered. This splitting, also known as zero-field splitting (ZFS) because of its presence even in the absence of an external magnetic field, is a large contribution to the fine structure of the rotational spectrum, and it can not be neglected even for qualitative simulations. From the computational point of view, the effect of the ZFS can be obtained from the calculation of two parameters, *D* and *E*. These are computed using the perturbation theory and account for two perturbations: the spin–spin dipole coupling and the second-order term of the spin–orbit coupling. The latter is usually negligible for small molecules, such as those under consideration, and it was not considered. As mentioned above, the computation has been carried out employing the MCSCF method in conjunction with the aug-cc-pVTZ basis set [39,49,50,51,59,60]. For MCSCF calculations, the complete active space chosen consists of all the valence electrons, i.e., twelve, in nine orbitals.

In passing we note that, in the following, we employ a short notation to indicate the electronic state under consideration: the molecular formulas are labelled with a superscript on the left side: 3 for the triplet and 1 for the singlet state.

## 3. Results

### 3.1. Molecular Structure

**Figure 1:**
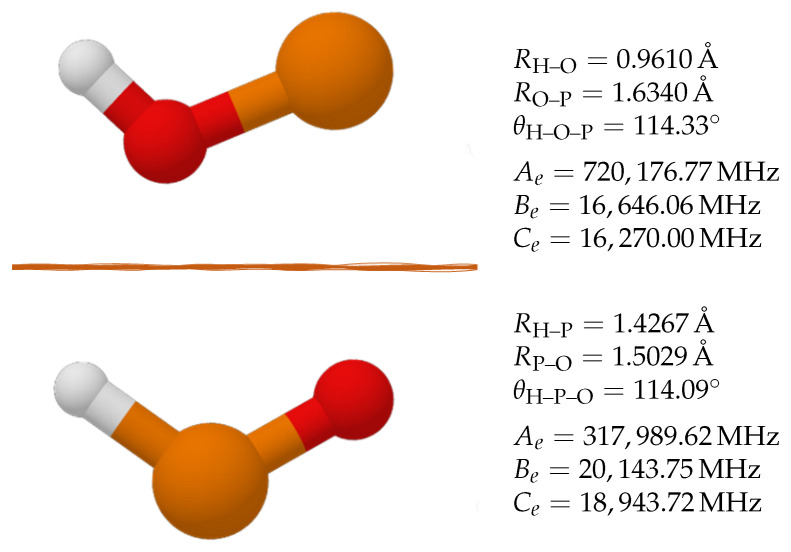
Structural parameters of ${}^{3}$POH and ${}^{3}$HPO along with the corresponding equilibrium rotational constants. Values at the “CC${}_{\mathrm{best}}$” level.

The electronic arrangement strongly affects the geometry of the molecule, and these changes are worth noticing, especially in small molecules, such as POH and HPO. Table 1 reports the molecular structure of POH and HPO in the electronic ground state and in the first excited state at the ae-CCSD(T)/cc-pwCVQZ level of theory. The inspection of Table 1 demonstrates that ${}^{1}$HPO has a P–O bond distance of 1.4799 Å, while the P–H bond length is 1.4521 Å. The excitation of one electron that leads to the triplet state strongly affects the molecular stability, with the P–O bond becoming longer by about 20 mÅ. At the same time, the P–H bond becomes shorter by about 30 mÅ. The angle $\theta_{H–P–O}$ varies from 104.5${}^{\circ }$ in the singlet state to 114.9${}^{\circ }$ in the first excited, triplet state. The remarkable changes noticed for HPO are mitigated in the POH system. In this case, the ground triplet state is characterised by a H–O bond distance of 0.9605 Å, and that of P–O is 1.6346 Å. The former bond length remains nearly unchanged in the singlet state, i.e., 0.9626 Å, while the P–O distance becomes shorter by 20 mÅ, similarly to what occurs in the HPO molecule. For comparison purposes, ${}^{3}$HPO and ${}^{3}$POH have been further investigated at the CC${}_{\mathrm{best}}$ level of theory and the corresponding geometries are reported in Figure 1. This higher level of theory corroborates the ae-CCSD(T)/cc-pwCVQZ results, with changes varying well within 2 mÅ for bond distances and 0.5${}^{\circ }$ for angles. The equilibrium rotational constants obtained from the CC${}_{\mathrm{best}}$ composite scheme are reported in Figure 1, and their accuracy will be discussed in Section 4 (Discussion).

A last note is deserved for the energy difference between ${}^{3}$POH and ${}^{3}$HPO. As already reported in the literature [27,28,30], the ${}^{3}$POH species is more stable than ${}^{3}$HPO. At the CC${}_{\mathrm{best}}$ level, such a difference is 93.7 kJ/mol. This stability slightly reduces when the zero-point energy (ZPE) is considered. Indeed, considering the harmonic ZPE at the ae-CCSD(T)/cc-pwCVQZ level of theory, the corrections amount to 23.7 kJ/mol for ${}^{3}$HPO and 33.6 kJ/mol for ${}^{3}$POH. This leads to a final energy difference between the two species of 83.8 kJ/mol. The anharmonic contribution, computed at the ae-CCSD(T)/cc-pwCVTZ level of theory, is less than 0.5 kJ/mol and does not change quantitatively the picture.

### 3.2. Spectral Simulations

The rotational spectrum of ${}^{3}$POH is rather complex due to the coupling of electronic and nuclear spin angular momenta. The electron spin (S=1) couples with the rotational angular momentum N and splits each rotational level with N>1 into three fine-structure sublevels labelled with the *J* quantum number. Each of these sublevels is further split by hyperfine interactions due to the nuclear spins of ${}^{31}$P and H, both having $I=\frac{1}{2}$. The angular momentum coupling scheme can be summarised as follows:

(6) $$J=N+S\;,\;F_{1}=J+I_{P}\;,\;F=F_{1}+I_{H}\;.$$

Here, F represents the total angular momentum, and J is the angular momentum excluding nuclear spins, whereas $I_{P}$ and $I_{H}$ are the phosphorus and hydrogen nuclear spins, respectively.

The rotational energies as well as transition frequencies and intensities can be derived using the effective Hamiltonian for an open-shell asymmetric rotor reported in Equation (5). In the present case, the term ${\tilde{H}}_{\mathrm{rot}}$ consists of the *S*-reduced Watson’s Hamiltonian [61] up to $N^{6}$ terms, whose coefficients are listed in Table 2. The fine- and hyperfine-structure terms (${\tilde{H}}_{\mathrm{fs}}$ and ${\tilde{H}}_{\mathrm{hfs}}$, respectively) are conveniently expressed using a spherical tensor notation [62]. The fine-splitting term includes energy contributions from electron spin–spin and electron spin–rotation interactions and can be written as [49,63]:

(7) $${\tilde{H}}_{\mathrm{fs}}=\frac{\sqrt{6}}{3}T^{2}\left(\lambda \right)\cdot T^{2}\left(S,S\right)+\frac{1}{2}\sum_{k}{\left[T^{k}\left(\epsilon \right),T^{k}\left(N,S\right)\right]}_{+}\;,$$

where *k* is the rank of the tensors, and the symbol ${\left[A,B\right]}_{+}$ represents the anticommutator of the *A* and *B* operators. The electron spin–spin coupling tensor λ is symmetric and traceless and is completely defined by the *D* and *E* constants reported in Table 3 through the following relations: $\lambda_{aa}=D$ and $\lambda_{bb}-\lambda_{cc}=E$. The electron spin-rotation tensor ϵ in turn has, for a planar asymmetric rotor, four cartesian non-vanishing elements: $\epsilon_{aa}$, $\epsilon_{bb}$, $\epsilon_{cc}$, and ${\tilde{\epsilon }}_{ab}=\frac{1}{2}\left(\epsilon_{ab}+\epsilon_{ba}\right)$ [32], for which the computed values are also reported in Table 3.

The hyperfine-structure term describes the interaction between the electron spin and the various non-zero nuclear spins present in the molecule [32]:

(8) $${\tilde{H}}_{\mathrm{hfs}}=\sum_{i}a_{F,i}T^{1}\left(I_{i}\right)\cdot T^{1}\left(S\right)+\frac{\sqrt{6}}{3}\sum_{i}T^{2}\left(T_{i}\right)\cdot T^{2}\left(I_{i},S\right)+\frac{1}{2}\sum_{k,i}{\left[T^{k}\left(C\right),T^{k}\left(N,I_{i}\right)\right]}_{+}\;,$$

where the index *i* runs over the ${}^{31}$P and H nuclei. The electron spin–nuclear spin coupling constants are the scalar Fermi-contact $a_{F}$ parameter and the elements of the dipolar coupling tensor $T_{\alpha \beta }$ (α,β=x,y,z). Small energy contributions due to the magnetic interaction of the nuclear spins with the molecular rotation are taken into account via the diagonal elements of the nuclear spin-rotation $C_{\alpha \alpha }$ tensor.

**Figure 2:**
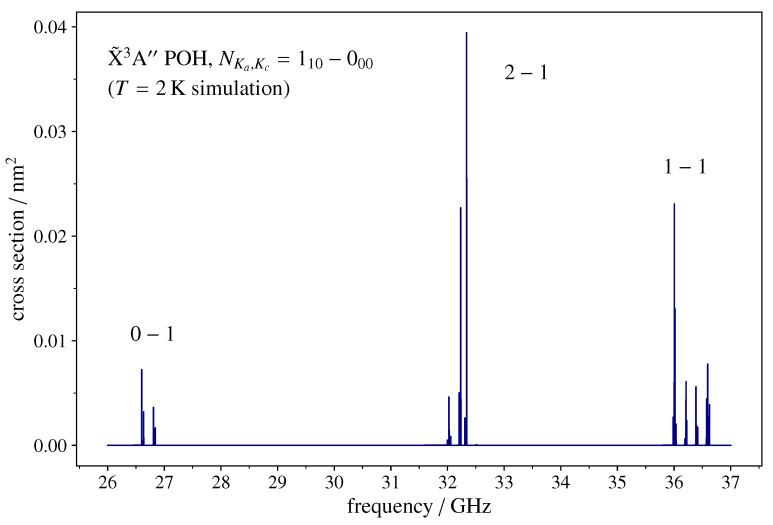
Spectral simulation of the $N_{K_{a},K_{c}}=1_{10}-0_{00}$ transition of ${}^{3}$POH in the $K_{a}$ microwave band, as can be observed using a broadband spectrometer. The fine-structure is a triplet of lines spread over ~10 GHz. Each $J^{\prime }\leftarrow J$ component is split in a number of closely spaced features by the hyperfine couplings. The *y*-axis scale indicates the absorption cross-section computed at 2 K, the typical rotational temperature obtained in a supersonic-jet cooled sample. See text for computational details.

The two non-zero components of the molecular electric dipole moment (see Table 2) generate *a*-type ($\Delta K_{a}=0,\Delta K_{c}=\pm 1$) and *b*-type ($\Delta K_{a}=\pm 1,\Delta K_{c}=\pm 1$) spectra. Each $N^{\prime }\leftarrow N$ transition typically consists of three widely separated $J^{\prime }\leftarrow J$ fine-structure lines, each split in many closely spaced $F_{1}^{\prime },F^{\prime }\leftarrow F_{1},F$ hyperfine components. An example of such a pattern is depicted in Figure 2, which shows the $N_{K_{a},K_{c}}=1_{01}-0_{00}$ rotational transition as it would be recorded by a broadband spectrometer operating in the $K_{a}$ microwave band. The rotational parameters employed for the simulations are reported in Table 2 and Table 3.

The rotational temperature has been set to 2 K, which corresponds to a partition function $Q_{\mathrm{spinrot}}=34.64$. The line shape is assumed to be a Gaussian function with a full width at half maximum (FWHM) of 500 kHz. These are typical values for the experimental conditions obtained in the supersonic expansion of a jet-cooled sample.

**Figure 3:**
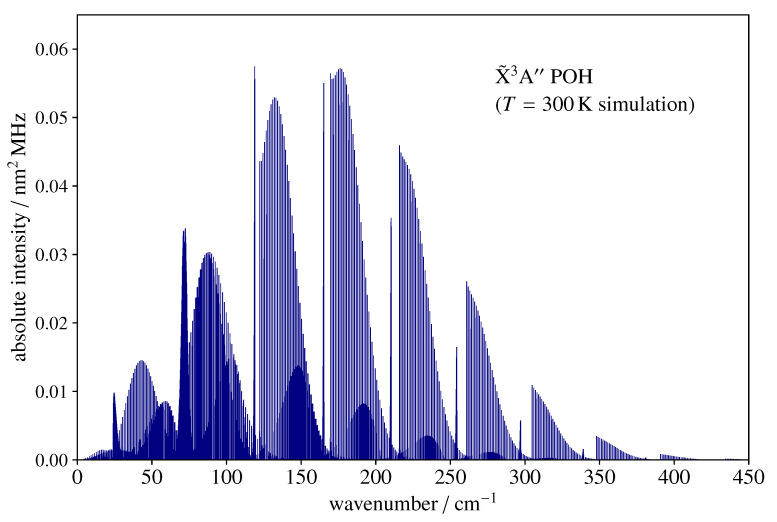
Far-infrared rotational spectrum (histogram) of ${}^{3}$POH computed at 300 K using the spectroscopic parameters of Table 2 and Table 3. The sharp-edge structures are produced by the *b*-type bands with $K_{a}+1\leftarrow K_{a}$ beginning at $K_{a}=N$. The *y*-axis scale indicates the absolute integrated intensity.

As POH is a relatively light molecule (B+C~39 GHz), its rotational spectrum at room temperature extends well into the far-infrared region (FIR). This is illustrated by Figure 3, which shows a stick spectrum simulation for ${}^{3}$POH in the wavenumber region up to 450 cm${}^{-1}$. The hyperfine interactions produce entirely negligible contributions in such a large energy span and have thus not been taken into consideration. The rotational temperature is assumed to be 300 K, and the corresponding partition function is $Q_{\mathrm{spinrot}}=5941.8$. The spectrum is prominently of *b*-type and is dominated by a number of $K_{a}+1\leftarrow K_{a}$ branches, which, as *N* increases, begins at increasingly large $K_{a}$, producing a noticeable sharp band head towards low frequencies.

**Figure 4:**
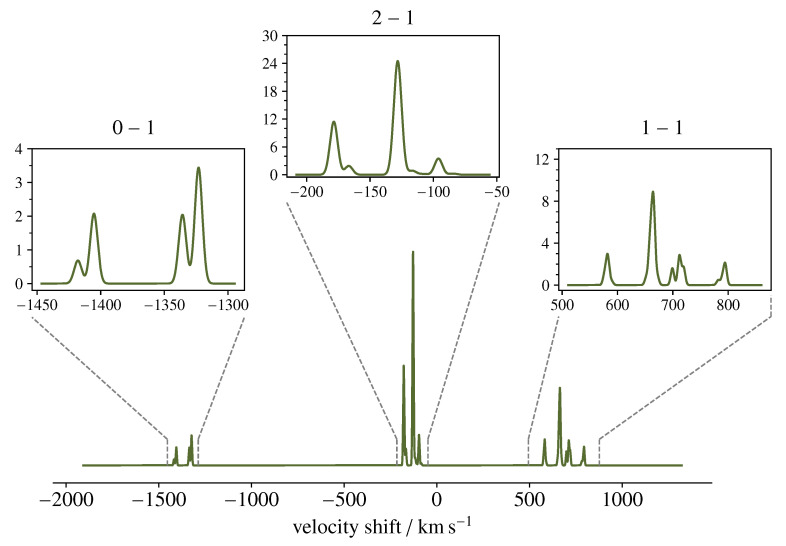
Simulation of the emission spectrum of the $N_{K_{a},\;K_{c}}=1_{11}-0_{00}$ transition of ${}^{3}$POH, as seen by the band 2b of the HIFI instrument onboard the former *Herschel* Space Telescope. The scale of the *x*-axis expresses the Doppler velocity shift Δν with respect to the hypothetically pure rotational transition frequency $\nu_{\mathrm{ref}}=751.778$ GHz: $\Delta \nu =c\;\left(\nu_{\mathrm{ref}}-\nu \right)/\nu_{\mathrm{ref}}$. The *y*-axis represents the main beam antenna temperature in units of mK. See text for computational details.

Due to the potential relevance of POH in astrochemistry, it is interesting to predict its spectrum in the typical interstellar conditions in order to figure out what are the most suited frequency intervals to aim for its detection and how its spectral features actually appear when targeted by one of the available observing tools operating in the millimetre-wave regime. For this simulation, we have assumed that POH may have a column density (*N*) and an excitation temperature ($T_{\mathrm{exc}}$) similar to that of PO observed towards the W51 star-forming complex [8], i.e., $N=4\times 10^{13}$ cm${}^{-2}$ and $T_{\mathrm{exc}}=35$ K. Owing to the relatively low temperature, only the low-lying rotational levels are populated enough to produce a detectable emission. In this context, one may ideally target the $N_{K_{a},K_{c}}=1_{11}-0_{00}$ line, whose upper level is located at 25 cm${}^{-1}$. This transition is centred at 752 GHz and thus covered by the 2b band of the HIFI instrument on board the former *Herschel* space telescope. Although this facility has discontinued operations, a wealth of data has been collected and stored in the ESA Herschel Science Archive (http://archives.esac.esa.int/hsa/whsa/) and can nowadays be explored when seeking for new molecular identifications [64]. The spectral simulation is shown in Figure 4. We assumed local thermodynamic equilibrium (LTE) conditions and a line FWHM of 7 km s${}^{-1}$, the same as exhibited by PO in W51 [8].

## 4. Discussion

The data obtained in this work can be compared with the little information already available in the literature for the system under consideration. As far as energetics are concerned, Ref. [27] reports an energy difference at equilibrium between ${}^{3}$POH and ${}^{3}$HPO of about 103 kJ/mol at the fc-CCSD(T)/aug-cc-pVQZ, which is in agreement with our CC${}_{\mathrm{best}}$ value of ~94 kJ/mol. Our energies can also be compared with the values provided in Ref. [30], which reported the investigation of the P + OH reaction using the so-called HEAT-like approach (see Table 1 of Ref. [30]), which is similar to our CC${}_{\mathrm{best}}$ level but also includes scalar-relativistic effects and the diagonal Born–Oppenheimer correction. The ${}^{3}$POH-${}^{3}$HPO energy separation at the equilibrium evaluated in Ref. [30] is about 99 kJ/mol, thus differing by about 5 kJ/mol. About 2–3 kJ/mol of this difference can be ascribed to the two missing terms in our approach, while the remaining discrepancy is probably due to the difference in the reference geometries, which are more accurate in our study (at the CBS+CV level). In fact, in Ref. [30], structures were optimised using a double-hybrid density functional.

Moving to structural considerations, the first comparison can be made with the data reported in Ref. [27]. In this latter work, for ${}^{3}$HPO, a $R_{H–P}$ distance of 1.431 Å, at the fc-CCSD(T)/aug-cc-pVQZ level, has been found, which compares well with our value of 1.424 Å (at the ae-CCSD(T)/cc-pwCVQZ level). Moving to the $R_{P–O}$ distance, this is predicted to be 1.499 Å by our calculations and is 11 mÅ shorter than in Ref. [27]. Concerning the angle, a discrepancy of only 0.3${}^{\circ }$ is noted. Our best computed values (CC${}_{\mathrm{best}}$) are the most accurate available in the literature and predict $R_{P–O}$ = 1.503 Å and $R_{H–P}$ = 1.427 Å. It is interesting to note that the CC${}_{\mathrm{best}}$ level of theory balances the effects of diffuse functions and core correlation, thus leading to equilibrium bond distances between the ae-CCSD(T)/cc-pwCVQZ and fc-CCSD(T)/aug-cc-pVQZ results. However, this does not apply to the angle. For the latter, the CC${}_{\mathrm{best}}$ value (114.1${}^{\circ }$) is slightly smaller than those obtained by the previous levels of theory. Moving to ${}^{3}$POH, at the ae-CCSD(T)/cc-pwCVQZ level, the angle is 113.9${}^{\circ }$, which is exactly the same value obtained with fc-CCSD(T)/aug-cc-pVQZ. However, the CC${}_{\mathrm{best}}$ level provides an angle of 114.3${}^{\circ }$, where the major contribution to this increase arises from the extrapolation to the CBS limit. The $R_{O–H}$ distance reported in Ref. [27] is identical to that obtained by the CC${}_{\mathrm{best}}$ approach, while the fc-CCSD(T)/aug-cc-pVQZ $R_{P–O}$ is 11 mÅ longer than our best value (1.634 Å).

A comment on the expected accuracy of the CC${}_{\mathrm{best}}$ results is deserved. According to the literature on this topic (e.g., Refs. [14,21,65]), bond distances are accurate to 1–2 mÅ and angles to 0.3${}^{\circ }$. However, the true assessment of the accuracy of the computed geometries can only be addressed in comparison with experimental data. As already mentioned, these do not exist for the triplet state of either POH and HPO. The only available experimental values are those for ${}^{1}$HPO [29], and they can compared with the theoretical estimates reported for the same species in Ref. [28], where the CBS+CV approach was used. Focusing on the rotational constant *B*, the experimental value is 21,075.835 MHz, whereas the equilibrium computed one is 21,166.10 MHz. This is in good agreement with the benchmark study of Ref. [65] that predicts an error of about 0.45% for the CC${}_{\mathrm{best}}$ equilibrium rotational constants ($B_{e}$) when compared with experimental $B_{0}$ values. According to this benchmark, an error of 0.2% is expected when computed and experimental ground-state rotational constants ($B_{0}$) are compared. Considering a vibrational contribution for ${}^{1}$HPO similar to that computed in the present work for ${}^{3}$HPO (i.e., ~0.6% of $B_{e}$), a $B_{0}$ value of 21,039.1 MHz is obtained for ${}^{1}$HPO, which is in agreement with the above statistical estimate. Similar evaluations can also be performed for the *A* and *C* rotational constants of ${}^{1}$HPO, thereby always obtaining uncertainties well within the maximum error estimate predicted by the benchmark study of Ref. [65], i.e., 1.2%. A similar accuracy is also expected for our ${}^{3}$POH and ${}^{3}$HPO results.

Finally, concerning the other spectroscopic parameters, there is very limited literature on the computation of the constants responsible for the fine structure. Instead, it is known that the ae-CCSD(T)/cc-pwCVQZ level of theory is able to provide quantitative estimates of the nuclear spin-rotation constants (see, e.g., Refs. [14,25,66,67]); the same applies to the scalar Fermi-contact $a_{F}$ parameter and the elements of the dipolar coupling tensor $T_{\alpha \beta }$ [68,69]. Therefore, despite the uncertainty of the absolute frequency scale, the predicted hyperfine structure of the rotational spectrum of ${}^{3}$POH can be used as a key pattern to identify this unstable species in laboratory experiments and in the interstellar medium.

## Figures and Tables

**Table 1:** Molecular structure of the singlet and triplet electronic states of HPO and POH as obtained at the ae-CCSD(T)/cc-pwCVQZ level of theory. Distances in Angstrom and angles in degrees.

Parameter	${}^{1}$HPO	${}^{3}$HPO	Parameter	${}^{1}$POH	${}^{3}$POH
$R_{H–P}$	1.4521	1.4244	$R_{H–O}$	0.9626	0.9605
$R_{P–O}$	1.4799	1.4989	$R_{O–P}$	1.6165	1.6346
$\theta_{H–P–O}$	104.54	114.88	$\theta_{H–O–P}$	111.79	113.92

**Table 2:** Computed rotational constants, centrifugal distortion parameters, and dipole moments of ${}^{3}$HPO and ${}^{3}$POH (Watson *S*-reduction, $I^{r}$ representation).

Parameter	Unit	${}^{3}$HPO	${}^{3}$POH
$A_{0}$	MHz	311,681.77	734,899.81
$B_{0}$	MHz	20,013.26	16,590.40
$C_{0}$	MHz	18,694.47	16,184.70
$D_{J}$	MHz	0.0247	0.0264
$D_{JK}$	MHz	2.57	2.20
$D_{K}$	MHz	127.26	509.0
$d_{1}$	kHz	−1.47	−0.53
$d_{2}$	kHz	−0.64	−0.06
$H_{J}$	Hz	−0.12	0.012
$H_{JK}$	kHz	0.051	0.004
$H_{KJ}$	kHz	−4.65	5.87
$H_{K}$	kHz	222.5	1666.20
$h_{1}$	mHz	11.3	0.81
$h_{2}$	mHz	9.24	0.93
$h_{3}$	mHz	5.79	0.12
$|\mu_{a}|$	D	2.45	0.65
$|\mu_{b}|$	D	0.51	1.39

**Table 3:** Computed fine and hyperfine parameters of ${}^{3}$HPO and ${}^{3}$POH.

Parameter	Unit	${}^{3}$HPO	${}^{3}$POH
*D*	MHz	9043.22	9503.08
*E*	MHz	1538.29	11.23
$\epsilon_{aa}$	MHz	604.16	−41.22
$\epsilon_{bb}$	MHz	−50.82	4.32
$\epsilon_{cc}$	MHz	−116.53	10.01
${\tilde{\epsilon }}_{ab}$	MHz	−461.71	−51.99
$a_{F}\left(P\right)$	MHz	759.78	115.90
$T_{aa}\left(P\right)$	MHz	−259.02	−617.40
$T_{bb}\left(P\right)$	MHz	−116.71	334.04
$T_{cc}\left(P\right)$	MHz	375.73	283.35
$T_{ab}\left(P\right)$	MHz	183.90	10.05
$C_{aa}\left(P\right)$	kHz	108.84	31.84
$C_{bb}\left(P\right)$	kHz	49.80	45.46
$C_{cc}\left(P\right)$	kHz	43.05	40.98
$a_{F}\left(H\right)$	MHz	196.92	24.64
$T_{aa}\left(H\right)$	MHz	−11.048	6.78
$T_{bb}\left(H\right)$	MHz	9.712	6.73
$T_{cc}\left(H\right)$	MHz	1.335	−13.51
$T_{ab}\left(H\right)$	MHz	−17.741	−20.36
$C_{aa}\left(H\right)$	kHz	−29.09	−64.52
$C_{bb}\left(H\right)$	kHz	2.03	−0.27
$C_{cc}\left(H\right)$	kHz	1.54	−2.08

## Data Availability

The data presented in this study are available upon request from the corresponding author.
